# Essential points from evidence-based clinical practice guideline for chronic kidney disease 2023

**DOI:** 10.1007/s10157-024-02497-4

**Published:** 2024-05-07

**Authors:** Japanese Society of Nephrology

**Affiliations:** Japanese Society of Nephrology, Nichinai-Kaikan 6F, 3-28-8 Hongo, Bunkyo-ku, Tokyo, 113-0033 Japan

## Introduction

### History of creation of this guideline

It has been clarified that chronic kidney disease (CKD) not only causes end-stage kidney disease but also increases the risk of developing cardiovascular disease (CVD) and death. There has been an increase in the number of chronic dialysis patients in Japan compared to that in other countries. As the population ages, an increase in CKD patients is expected; therefore, this is a particularly important issue.

To date, clinical practice guidelines or guides have played a major role in the dissemination, public awareness, and practice of CKD treatment. In 2002, a guideline on CKD diagnosis and management was published by the National Kidney Foundation Kidney Disease Outcomes Quality Initiative (NKF KDOQI). Since then, Kidney Disease: Improving Global Outcomes (KDIGO) has taken over the creation of guidelines related to CKD. In Japan, the Japanese Society of Nephrology published the “Clinical Practice Guidebook for Diagnosis and Treatment of Chronic Kidney Disease” in 2007 as a tool for deepening collaboration between primary care physicians and nephrologists as well as facilitating collaboration between hospitals and clinics, which was revised in 2009 and 2012. This guidebook was aimed at primary care physicians and includes expert opinions. The “Evidence-based Clinical Practice Guideline for CKD” was published in 2009 with specialist physicians as the primary users and laid further emphasis on evidence during the guideline’s creation process. This guideline had been certified as an appropriate clinical practice guideline by a Japanese third-party organization that evaluates the creation process and contents (Japan Council for Quality Health Care). Afterwards, the “Evidence-based Clinical Practice Guideline for CKD 2013” was published, where the guideline creation process was made more rigorous, and a clinical question (CQ) style was adopted throughout the guideline. The results of questionnaire surveys conducted after publication clarified that the clinical practice guideline can be an important decision-making tool for non-specialist primary care physicians rather than nephrologists. Based on this, “Evidence-based Clinical Practice Guideline for CKD 2018” was created in a completely revised form, with the assumed users as not only specialist physicians, but also as non-specialist primary care physicians. The same revision committee created the “CKD Medical Care Guide for Patients and Families 2018” with the assumed users as patients and their families.

Here, in creation of the “Evidence-based Clinical Practice Guideline for CKD 2023”, two aspects have been changed from the previous version. The first is that this version is not necessarily limited to the CQ format. Though this format is useful, there was dilemma regarding its use for describing important content related to CKD treatment, and we were unable to effectively communicate our message to the reader. To overcome such a situation in this revision, we created items and textual commentary that included CQs and expert opinions, respectively. The second is that we decided to create a “Clinical Practice Guidebook for Diagnosis and Treatment of Chronic Kidney Disease” following this guideline. Several primary care physicians had suggested that the clinical practice guidelines included a large amount of information and something easier to use would be preferred by them. Therefore, following this recommendation, the same committee decided to revise a “Clinical Practice Guidebook for Diagnosis and Treatment of Chronic Kidney Disease,” which had not been revised since 2012, for use by primary care physicians and medical staff. Furthermore, we plan to create a revised version of the “CKD Medical Care Guide for Patients and Families” for use by patients and their families in 2024, which will further clarify the position of the “Evidence-based Clinical Practice Guideline for CKD 2023”.

We hope that the practice of medical care based on this guideline will lead to the early detection of CKD, promotion of cooperation between primary care and specialist physicians, prevention of CKD progression, and not only reduction in the onset of CVD and initiation of dialysis, but also in promotion of public health.

Strength of evidence and recommendations for general outcome

[Strength of recommendation (recommendation level)]

1: We recommend

2: We suggest

[Strength of evidence for general outcome (evidence grade)]

A (Strong): strong confidence level in estimated effect

B (Medium): moderate confidence level in estimated effect

C (Weak): limited confidence level in estimated effect

D (Extremely weak): hardly any confidence in estimated effect

None

## Summary of CQs and statements

### Chapter 1. CKD diagnosis and its clinical significance

#### 1-1. CKD diagnosis (Table 1)

[Commentary summary]

The definition of CKD is as follows, and a diagnosis is made when either (1) or (2) or both persist for over three months.

1. Abnormal urinalysis, diagnostic imaging, blood tests, or pathological diagnosis clearly indicative of kidney injury; presence of proteinuria of 0.15 g/gCr or more (albuminuria of 30 mg/gCr or more) is particularly important.
2. GFR < 60 mL/min/1.73 m^2^

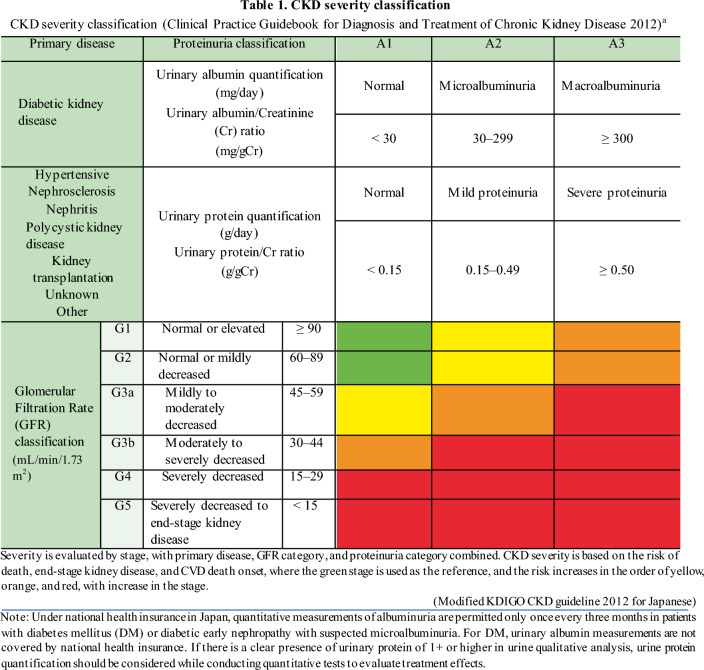


#### 1-2-1 CKD severity evaluation method: evaluation of kidney function

[Commentary summary]

Kidney function is evaluated using GFR. In daily clinical practice, the serum Cr level, sex, and age are used in calculation using the Japanese GFR estimation formula (JSN eGFRcr). The serum cystatin C level-based Japanese GFR estimation formula (JSN eGFRcys) is used as needed. GFR measurement by inulin clearance is used when a more accurate evaluation of kidney function is needed.

JSN eGFRcr:

- Male 194 × Serum Cr (mg/dL)^−1.094^ × age (years)^−0.287^ (mL/min/1.73 m^2^).
- Female 194 × Serum Cr (mg/dL)^−1.094^ × age (years)^− 0.287^ × 0.739 (mL/min/1.73 m^2^).
- Note: The Cr value (notation to two decimal places) measured by the enzymatic method is used.

JSN eGFRcys:

- Male 104 × Serum cystatin C (mg/L)^−1.019^ × 0.996^age (years)^ − 8 (mL/min/1.73 m^2^).
- Female 104 × Serum cystatin C (mg/L)^−1.019^ × 0.996^age (years)^ × 0.929 − 8 (mL/min/1.73 m^2^).
- These estimation formulas apply to those aged 18 years and older.

#### 1-2-2 CKD severity evaluation method: evaluation of proteinuria and albuminuria

[Commentary summary]

1. Proteinuria/albuminuria is a strong risk factor for serious events such as end-stage kidney disease, CVD death, and all-cause death, and is an important diagnostic item for CKD. Evaluation for proteinuria or albuminuria is essential at the time of CKD diagnosis and grading.
2. Proteinuria and albuminuria are criteria for conducting kidney biopsy to detect the primary disease. Kidney biopsy is considered if proteinuria > 0.5 g/day or if the test is positive for both proteinuria and hematuria.
3. Proteinuria/albuminuria is useful as a risk evaluation, treatment effect, and treatment selection index during CKD follow-up observations and treatment, and it should be evaluated regularly.
4. Treatment interventions that are expected to reduce proteinuria and albuminuria and have a reno-protective effect include salt intake and weight reduction, and multi-faceted intervention for CKD risk factors. Pharmacotherapy using renin–angiotensin (RA) system inhibitors, mineralocorticoid receptor antagonists, sodium-glucose cotransporter-2 (SGLT2) inhibitors, etc. is useful.

#### 1-2-3 CKD severity evaluation method: evaluation of cause

[Commentary summary]

CKD is a condition that encompasses various kidney diseases; treatment, kidney function, and vital prognosis differ depending on the cause. In particular, nephritis is a pathological condition that requires specialized diagnosis and treatment; the histological diagnosis determines the treatment modality to be followed. The clinical course is also different for diabetes mellitus (DM) and hypertension-related kidney disease. The cause, GFR, and albuminuria (CGA) classification severity, which is GFR- and urinary albumin-based severity combined with the cause of kidney disease, better reflects the prognosis. Based on the above, the cause of CKD should be investigated with its diagnosis, and the severity should be recorded.

#### 1-3 CKD progression evaluation

[Commentary summary]

1. We evaluate risk according to stage of the CGA classification of CKD and other comorbidities and risk factors for end-stage kidney disease. Furthermore, significant predictors of end-stage kidney disease are a doubling of the serum Cr level (equivalent to 57% decrease in estimated glomerular filtration rate (eGFR)), and a 40% or 30% decrease in eGFR over a 1–3-year period. These factors can also be indicators for CKD progression.
2. The eGFR slope is a useful predictor for renal prognosis, and rapid progression is defined as a slope more negative than − 5.0 mL/min/1.73 m^2^/year.
3. Changes in the eGFR slope may also serve as a surrogate endpoint for end-stage kidney disease and an indicator of CKD progression.
4. There is usually a decrease in eGFR in the early stages of RA system and SGLT2 inhibitor administration; however, a nephrologist should be referred to in case a decrease of 30% or more is observed within three months.

#### 1-4-1 CKD patient referral criteria for recommending medical examination recipients to visit medical institutions

[Commentary summary]

Visiting a medical institution is recommended in the following circumstances-

1. If urinary protein is (1+) or higher.
2. If urinary protein ( ±) is observed for two consecutive years.
3. If eGFR is less than 45 mL/min/1.73 m^2^ (CKD stage G3b or later). For those under the age of 40 years, if eGFR is less than 60 mL/min/1.73 m^2^ (CKD stage G3a).

#### 1-4-2 CKD patient referral criteria for referral from primary care physician to nephrologist/specialized medical institution

[Commentary summary]

Referral from primary care physician to nephrologist/specialized medical institution should be done in the following cases:

1. At CKD stage G1 and G2, patients with hematuria at proteinuria classification A2 or A3; patients without hematuria at proteinuria classification A3.
2. At CKD stage G3a, those aged 40 years or older and at proteinuria classification A2 or A3; those aged less than 40 years, regardless of their proteinuria classification.
3. At CKD stage G3b–G5, regardless of their proteinuria classification.
4. If kidney function deteriorates by 30% or more within three months.

### Chapter 2. Hypertension/CVD (heart failure)

#### 2-1 Blood pressure management to suppress CKD onset in hypertensive patients (Tables 2 and 3)

[Commentary summary]

Multiple observational studies have shown that hypertension is a risk factor for CKD onset. Therefore, preventing CKD onset through blood pressure management is important in terms of improving vital prognosis and reducing medical costs. However, few interventional studies for hypertension have examined CKD incidence, and evidence for this topic is insufficient. Based on the above, although the specific management goals for suppressing the onset of CKD are unknown, the benefit seems to be clear based on a number of observational studies, and in antihypertensive treatment for hypertensive patients without CKD, it is desirable to implement blood pressure management, including diet and exercise therapy. This is based on the general antihypertensive target values recommended in the Japanese Society of Hypertension Guidelines for the Management of Hypertension (JSH 2019).

#### 2–2 [CQ] Is antihypertensive therapy to reduce office blood pressure below 130/80 mmHg recommended for CKD patients with hypertension?

[Recommendation]

 <CKD stage G1, G2>

DM (+): < 130/80 mmHg recommended. [1B]

DM (−): For proteinuria classification A1, < 140/90 mmHg recommended. [1A]

DM (−): For proteinuria classification A2 or A3, < 130/80 mmHg recommended. [1C]

 <CKD stage G3–G5>

DM (+): < 130/80 mmHg suggested. [2C]

DM (−): For proteinuria classification A1, < 140/90 mmHg (< 130/80 mmHg should be judged on an individual basis after considering the balance between benefits and harms) suggested. [2C]

DM (−): For proteinuria classification A2 or A3, < 130/80 mmHg suggested. [2C]

In either case, it is suggested that appropriate blood pressure management be conducted with attention to hypotension and dizziness accompanying the strengthening of antihypertensive management. [2C]
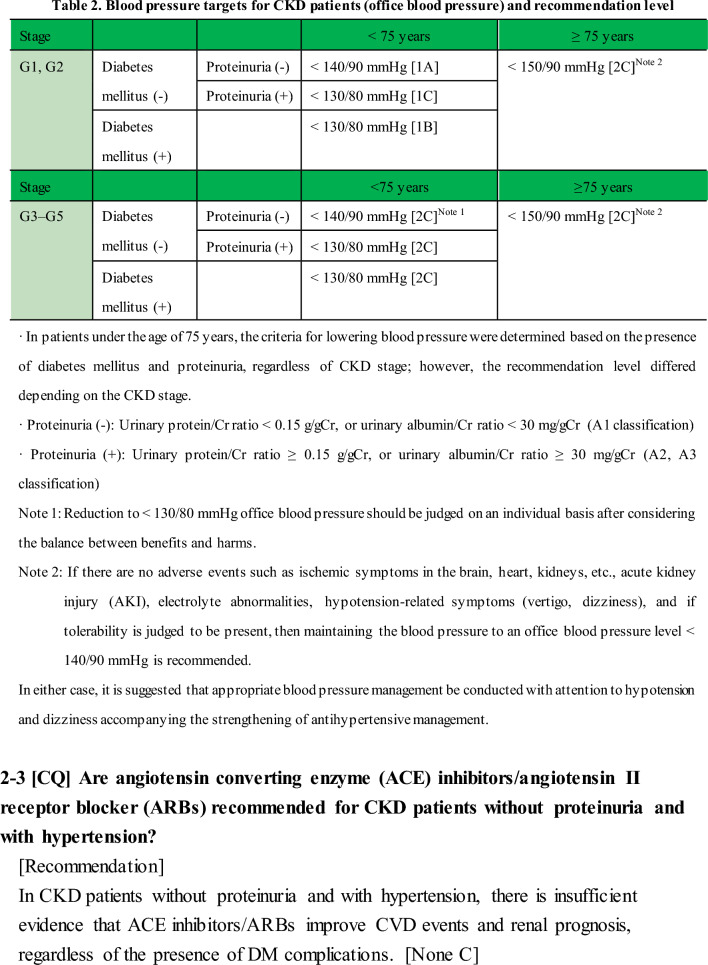


#### 2-3 [CQ] Are angiotensin converting enzyme (ACE) inhibitors/angiotensin II receptor blocker (ARBs) recommended for CKD patients without proteinuria and with hypertension?

[Recommendation]

In CKD patients without proteinuria and with hypertension, there is insufficient evidence that ACE inhibitors/ARBs improve CVD events and renal prognosis, regardless of the presence of DM complications. [None C]
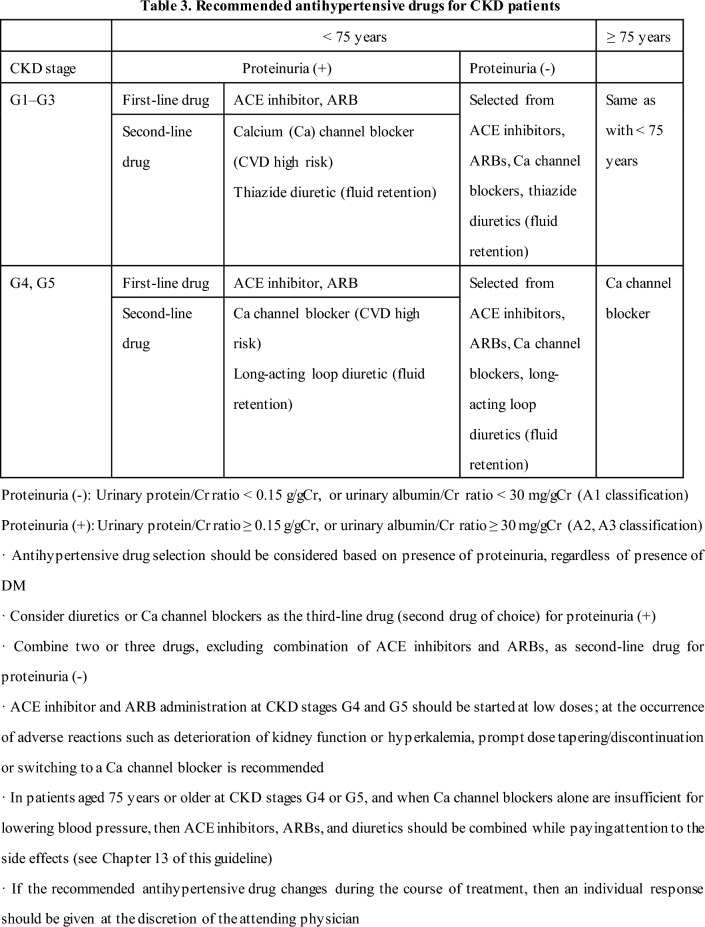


#### 2-4 [CQ] Which therapeutic agents are recommended for CKD patients complicated with heart failure?

[Recommendation]

The strength of evidence for the recommended therapeutic drug differs depending on the CKD stage and drug type. Therefore, examining their use while considering the risks and benefits is recommended in CKD stage G4 and G5 (see the Table 4 for the recommended classes and evidence levels).
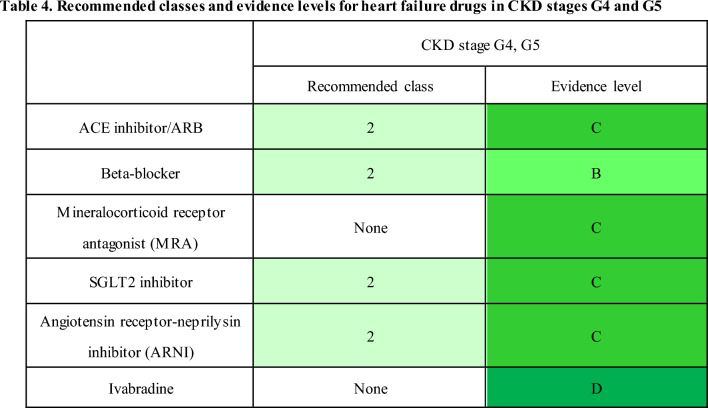


### Chapter 3. Hypertensive nephrosclerosis/renal artery stenosis

#### 3-1 [CQ] Are renin–angiotensin–aldosterone system (RAAS) inhibitors recommended more than other antihypertensive drugs for CKD with renal artery stenosis?

[Recommendation]

For cases of CKD with unilateral renal artery stenosis, RA system inhibitors may reduce progression to end-stage kidney disease and mortality risk compared to other antihypertensive drugs, and their use is suggested. [2C]

However, due to the risk of AKI onset, treatment should be started with a low dose and the dose should be carefully adjusted while monitoring the serum Cr and potassium (K) levels. [None D]

In cases with suspected bilateral renal artery stenosis, these drugs should not be used as a general rule. [None D]

#### 3-2 Hypertensive nephrosclerosis diagnosis and treatment

[Commentary summary]

Hypertensive nephrosclerosis is a renal lesion caused by persistent hypertension. It generally refers to benign nephrosclerosis. Cases with a clinical history of hypertension; absence of hematuria and proteinuria; and kidney dysfunction without diabetes mellitus, primary glomerulonephritis, or secondary glomerulonephritis, are often diagnosed as hypertensive nephrosclerosis. Even if hypertension is not present at the time of diagnosis, kidney biopsy may show nephrosclerosis, suggestive of the effects of previous hypertension, aging, and ischemia.

Blood pressure management is important for treatment. In addition to the retarding kidney function deterioration, subsequent onset of CVD is often observed; therefore, treatment should be considered from the perspective of suppressing CVD progression. Antihypertensive goals and first-line drugs are based on Chapter 2 of this guideline.

#### 3-3 [CQ] Is revascularization recommended for CKD with atherosclerotic renal artery stenosis?

[Recommendation]

For cases of CKD with atherosclerotic renal artery stenosis, revascularization does not reduce the risk of kidney injury progression, CVD, or death. Therefore, this treatment is generally not suggested, considering the risk of complications. [2B]

However, revascularization may be considered in patients with treatment-resistant hypertension. [None D]

#### 3-4 Imaging tests for renal artery stenosis

[Commentary summary]

Renal artery ultrasound tests should be conducted as screening tests, followed by non-contrast magnetic resonance (MR) angiography as a next step. While conducting computed tomography (CT) angiography or gadolinium-enhanced (Gd-enhanced) MR angiography, there is a need to sufficiently consider the risks of contrast-enhanced nephropathy and nephrogenic systemic fibrosis. Selective digital subtractive angiography (DSA) is conducted if these tests do not lead to a diagnosis or in case angioplasty is indicated.

### Chapter 4. Diabetic kidney disease (Fig. 1)

**Fig. 1:**
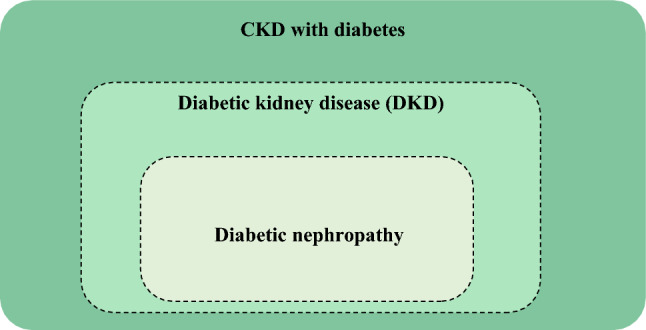
Conceptual diagram of DKD. DKD is a concept that includes not only typical diabetic nephropathy, but also atypical diabetes-related kidney disease in which eGFR declines without prominent albuminuria. Furthermore, CKD with diabetes mellitus is a broader concept that includes patients with kidney disease, not directly associated with diabetes mellitus [e.g., IgA nephropathy, polycystic kidney disease (PKD)], but who are comorbid with diabetes mellitus. DKD and diabetic nephropathy are clearly classified according to CKD severity classification and staging of diabetic nephropathy; however, the involvement of diabetes mellitus may be difficult to infer without kidney biopsy. Hence, the interfaces in the diagram are indicated using dashed lines

DKD is a concept that includes not only typical diabetic nephropathy, but also atypical diabetes-related kidney disease in which eGFR declines without prominent albuminuria. Furthermore, CKD with diabetes mellitus is a broader concept that includes patients with kidney disease, not directly associated with diabetes mellitus [e.g., IgA nephropathy, polycystic kidney disease (PKD)], but who are comorbid with diabetes mellitus. DKD and diabetic nephropathy are clearly classified according to CKD severity classification and staging of diabetic nephropathy; however, the involvement of diabetes mellitus may be difficult to infer without kidney biopsy. Hence, the interfaces in the diagram are indicated using dashed lines.

#### 4-1 [CQ] Is urinary albumin measurement recommended for patients with DKD?

[Recommendation]

Regular urinary albumin measurement in patients with DKD is useful for predicting a prognosis and is strongly recommended. [1B]

#### 4-2 [CQ] Is the use of diuretics (e.g., loop diuretics, thiazide diuretics, mineralocorticoid receptor antagonists) recommended for patients with DKD?

[Recommendation]

There is insufficient evidence supporting the prevention of DKD progression by the use of loop and thiazide diuretics. [No recommendation]

The use of mineralocorticoid receptor antagonists is suggested as it may show improvement in urinary albumin in DKD patients. [2C]

The use of loop diuretics is suggested in DKD patients with fluid overload. [2D]

#### 4-3 [CQ] Is glycemic control to HbA1c < 7.0% recommended for patients with DKD and macroalbuminuria?

[Recommendation]

A uniform recommendation for patients with DKD and macroalbuminuria is difficult; glycemic control to HbA1c < 7.0% is suggested as a target value to suppress the onset and progression of microvascular complications. However, the goal of blood glucose levels in patient should be individualized according to their clinical background. [2C]

#### 4-4 [CQ] Is intensive treatment recommended for diabetic patients to prevent the onset and progression of DKD?

[Recommendation]

Intensive treatment is recommended in patients with diabetes because it is expected to suppress the progression of DKD and albuminuria. [1A]

#### 4-5 [CQ] Is SGLT2 inhibitor administration recommended for patients with DKD?

[Recommendation]

SGLT2 inhibitor use is recommended in patients with DKD because it is expected to improve renal prognosis and suppress the onset of CVD. [1A]

### Chapter 5. Dyslipidemia/hyperuricemia

#### 5-1 [CQ] Is urate-lowering therapy recommended for pre-dialysis CKD patients?

[Recommendation]

Urate-lowering therapy in CKD patients with hyperuricemia may suppress the deterioration of kidney function, and may be considered as a therapy. [2C]

#### 5-2 [CQ] Is lipid-lowering therapy recommended for pre-dialysis CKD patients?

[Recommendation]

Lipid-lowering therapy with statins and statin-ezetimibe concomitant therapy for CKD patients with dyslipidemia may suppress the onset of CVD events and deterioration of kidney function, and is suggested as a therapy. [2B]

Lipid-lowering therapy with fibrates for CKD patients with dyslipidemia may be useful in suppressing the onset of CVD events; however, in patients with moderate to severe kidney injury, fibrates require careful administration or are contraindicated; therefore, caution is required for the therapy. [None D]

### Chapter 6. Lifestyle habits

#### 6-1 Smoking cessation in patients with CKD

[Commentary summary] The interventional effect of smoking cessation in patients with CKD is unclear; however, it is strongly recommended for these patients, along with the general population.

#### 6-2 Alcohol consumption in patients with CKD

[Commentary summary] There is insufficient evidence to show the effect of alcohol consumption on progression of CKD and death in patients.

#### 6-3 Effect of suppressing the progression of CKD due to coffee intake

[Commentary summary] Coffee intake is expected to suppress the progression of CKD.

#### 6-4 Oral care in patients with CKD

[Commentary summary] Unhealthy oral cavity conditions gradually worsen with progression of CKD stage. An association between frailty and increased mortality has been suggested. Hence, oral care is recommended for patients with CKD as well.

#### 6-5 Constipation in patients with CKD

[Commentary summary] Constipation may be a risk factor for the onset and progression of CKD.

#### 6-6 Measures for COVID-19 infection prevention in patients with CKD

[Commentary summary] CKD is an aggravating factor for COVID-19 and infection prevention measures are particularly important.

#### 6-7 [CQ] Is intentional increase in water intake recommended in pre-dialysis CKD patients?

[Recommendation]

In pre-dialysis patients, improvements in vital prognosis and reno-protective effects cannot be expected with increased water intake. Therefore, intentional increase in water intake is not suggested. [2B]

#### 6-8 [CQ] Is securing an adequate amount of sleep recommended for patients with CKD?

[Recommendation]

In patients with CKD, adequate sleep may reduce the possibility of dialysis initiation and CVD onset. Hence, securing an adequate amount of sleep is suggested. [2D]

#### 6-9 [CQ] Is exercise recommended in pre-dialysis patients with CKD who are not obese?

[Recommendation]

In pre-dialysis patients with CKD who are not obese, daily exercise does not increase proteinuria and may improve kidney function and physical quality of life (QOL). Hence, exercising to a possible extent, while considering complications and physical function, including cardiopulmonary function, is suggested. [2C]

#### 6-10 [CQ] Is vaccination recommended for pre-dialysis patients with CKD?

[Recommendation]

In pre-dialysis patients, vaccinations against hepatitis B, influenza viruses, and pneumococcal infections are strongly recommended as preventive measures against infectious diseases. [1D]

#### 6-11 [CQ] Is multidisciplinary educational intervention on lifestyle habits recommended for adult pre-dialysis patients with CKD?

[Recommendation]

Multidisciplinary educational interventions for adult pre-dialysis patients with CKD may have a suppressive effect on decreased kidney function and reduce the onset of CVD events. Therefore, conducting sessions on these interventions is preferred. [2C]

### Chapter 7. Progression of CKD and obesity/metabolic syndrome

#### 7-1 [CQ] Are lifestyle interventions (diet therapy/exercise therapy) recommended for CKD patients with obesity or metabolic syndrome (MetS)?

[Recommendation]

Lifestyle interventions (diet therapy/exercise therapy) for CKD patients with obesity or MetS are potentially effective in reducing albuminuria/proteinuria and preventing eGFR decline. [2C]

However, there may be individual differences in age, comorbidities, lifestyle backgrounds, values, preferences, and tolerability. Therefore, individual judgments on the method and degree are needed. [None D]

#### 7-2 Impact of obesity/metabolic syndrome (MetS) on mortality, cardiovascular, and kidney prognoses in patients with CKD

[Commentary summary]

While obesity is not a clear risk factor for mortality, CVD, or CKD progression, in patients with CKD, MetS may be a risk factor. However, it should be noted that this evaluation is based mainly on the results of observational or cohort studies.

#### 7-3 Usefulness of bariatric and metabolic surgery on mortality, cardiovascular, and kidney prognoses in obese patients with CKD

[Commentary summary]

Bariatric and metabolic surgery may reduce the risk of not only CKD progression, but also all-cause mortality and CVD onset in obese patients with CKD.

### Chapter 8. Nutrition

#### 8-1 [CQ] Is intervention by registered dietitians recommended in the care of patients with CKD?

[Recommendation]

Intervention by registered dietitians in the care of patients with CKD is recommended because they may suppress CKD stage progression and delay the initiation of renal replacement therapy. [1C]

#### 8-2 [CQ] Is protein intake restriction recommended in patients with CKD?

[Recommendation]

It is recommended that the required energy intake and restricted protein intake be maintained under the supervision of a medical team, including a nephrologist and dietitian, since this is expected to suppress CKD stage progression. [1B]

#### 8-3 [CQ] Is the management of serum K levels recommended in patients with CKD?

[Recommendation]

It is recommended that serum K levels in patients with CKD be managed between 4.0 and 5.5 mEq/L, as this may reduce the risk of all-cause mortality and CVD. [1C]

#### 8-4 [CQ] Is salt restriction recommended for patients with CKD?

[Recommendation]

Salt restriction of less than 6 g/day is recommended because this will reduce the blood pressure and urinary protein parameters in patients with CKD. [1C]

However, its effects on progression to end-stage kidney disease, all-cause mortality, and CVD events are unknown. [None D]

#### 8-5 [CQ] Are dietary interventions for metabolic acidosis in patients with CKD recommended?

[Recommendation]

In CKD patients with metabolic acidosis, dietary intervention with alkaline foods (e.g., vegetables and fruits) is suggested because it may suppress net endogenous acid production and prevent deterioration of renal function. [2C]

### Chapter 9. Renal anemia

#### 9-1 [CQ] What is the appropriate target hemoglobin (Hb) level for erythropoiesis stimulating agent (ESA) therapy in CKD patients with renal anemia?

[Recommendation]

It is recommended not to aim for an Hb level of 13 g/dL or more when administering ESA for renal anemia in pre-dialysis CKD patients. [2B]

There is insufficient underlying evidence; however, it is suggested that the lower limit of the target Hb level be 10 g/dL as a guideline, and that decisions be made according to QOL, background factors, and pathological condition of individual cases. [None D]

#### 9-2 [CQ] Is iron administration recommended for CKD patients with anemia?

[Recommendation]

Iron administration is recommended if iron deficiency is present in CKD patients with anemia. [2B]

#### 9-3 Addition to “Recommendation for appropriate use of HIF-prolyl hydroxylase (HIF-PH) inhibitors (September 29, 2020 version)”

[Commentary summary]

The Japanese Society of Nephrology has published the “Recommendation for appropriate use of HIF-PH inhibitors (September 29, 2020 version)”. This recommendation has been published recently and does not have major changes in its summary. Practitioners should refer to the “Recommendation for appropriate use of HIF–PH inhibitors” (2020).

### Chapter 10. CKD-MBD

#### 10-1 [CQ] Is phosphate-lowering therapy recommended for pre-dialysis patients with CKD?

[Recommendation]

The use of phosphate binders is suggested in patients with hyperphosphatemia because it may reduce the risk of progression to kidney failure. [2C]

The effect of a phosphate-restricted diet on survival is not clear. [None D]

#### 10-2 [CQ] Are calcium (Ca)-free phosphate binders recommended when prescribing phosphate binders to pre-dialysis patients with CKD?

[Recommendation]

The use of Ca-free phosphate binders is suggested for the treatment of hyperphosphatemia in pre-dialysis patients with CKD because it may reduce the risk of death, kidney failure, and progression of vascular calcification compared with Ca-containing phosphate binders. [2C]

#### 10-3 [CQ] Is the prescription of active vitamin D recommended for pre-dialysis patients with CKD?

[Recommendation]

The use of active vitamin D in pre-dialysis patients with CKD may be considered on a case-by-case basis. [2C]

However, it is suggested that the dose of active vitamin D be reduced or discontinued if hypercalcemia develops. [2D]

#### 10-4 [CQ] Is pharmacotherapy for osteoporosis recommended in pre-dialysis CKD patients with osteoporosis?

[Recommendation]

In pre-dialysis CKD patients with osteoporosis (CKD stage G3a or G3b), pharmacotherapy for osteoporosis may reduce the risk of fracture compared with no treatment. Therefore, it is suggested that treatment be carefully implemented with attention to drug-specific side effects. [2C, 2D]

It should be noted that the strength of evidence varies for each class of drug (Table 5).

There is insufficient evidence to support the use of osteoporosis medications in patients with CKD stages G4 and G5. The risks and benefits should be considered on a case-by-case basis. [None D]

Osteoporosis treatment for patients with CKD stages G1 and G2 can be equivalent to the general population and should follow the “Japanese 2015 Guidelines for Prevention and Treatment of Osteoporosis”.
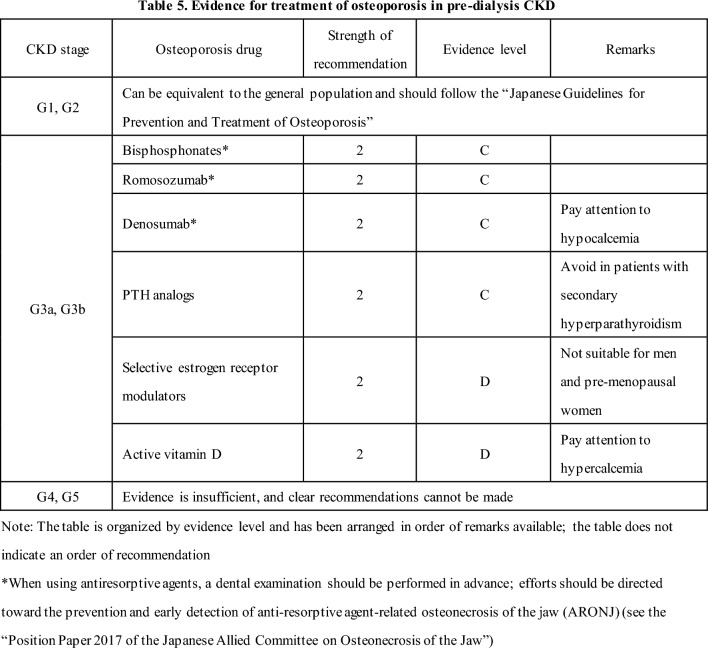


### Chapter 11 Pharmacotherapy

#### 11-1 [CQ] Is the use of spherical carbon adsorbent recommended for patients with CKD?

[Recommendation]

The effects of administration of spherical carbon adsorbent to patients with CKD on preventing hard endpoints such as progression to end-stage kidney disease and death are unclear; however, it may delay the rate of decrease in kidney function, and its use may be considered. [2C]

#### 11-2 [CQ] Is sodium bicarbonate administration recommended for CKD patients with metabolic acidosis?

[Recommendation]

In pre-dialysis cases of CKD with metabolic acidosis (CKD stages G3–G5), interventions with sodium bicarbonate may suppress the deterioration of kidney function, and it is suggested that they be conducted while paying attention to the exacerbation of edema. [2B]

#### 11-3 [CQ] Is SGLT2 inhibitor administration recommended for CKD patients without diabetes mellitus?

[Recommendation]

SGLT2 inhibitor administration in CKD patients without diabetes mellitus and with proteinuria is expected to suppress the progressive deterioration of kidney function and suppress the onset of CVD events and death. Hence, its administration is recommended. [1B]

There is no evidence for improvement in the condition of patients without proteinuria or those with an eGFR of less than 20 mL/min/1.73 m^2^ by the initiation of SGLT2 inhibitor therapy. [None D]

#### 11-4 [CQ] Is the discontinuation of RA system inhibitors recommended for CKD stage G4 and G5 patients?

[Recommendation]

It is suggested that RA system inhibitors in use should not be uniformly discontinued for CKD stages G4 and G5 as there is a possibility that the vital prognosis may worsen, although its impact on the renal replacement therapy (RRT) risk is unclear. [2C]

#### 11-5 [CQ] Is long-term concomitant use of proton pump inhibitors (PPIs) for the treatment of gastrointestinal ulcers, reflux esophagitis, or for the prevention of recurrence of those diseases under the administration of low-dose aspirin a risk of CKD onset and progression?

[Recommendation]

Long-term concomitant use of PPIs may be a risk factor for CKD onset and progression. It is suggested that PPIs be used only when they are therapeutically necessary. [2C]

#### 11-6 Kidney function estimation formula used for setting drug doses by kidney function (Table 6)

[Commentary summary]

- As a general rule for the patient kidney function estimation formula, the kidney function evaluation method (at the time of the clinical trial) that is used to set drug doses by kidney function in the package insert should be used. However, eGFR should be used for the patient’s kidney function when the dose setting by kidney function is eCcr (Jaffe method).
- In patients with non-standard body sizes (e.g., sarcopenia or obesity), renal function assessment methods appropriate for the patients’ body size should be used.
- The drug dose setting by kidney function should be used as a standard, and avoiding overdose or underdose is important. For patients treated by high-risk drugs and those with special physiques, careful evaluations of kidney function should be conducted, and doses taking into consideration of the physique and pathophysiological condition in individual patients should be set.

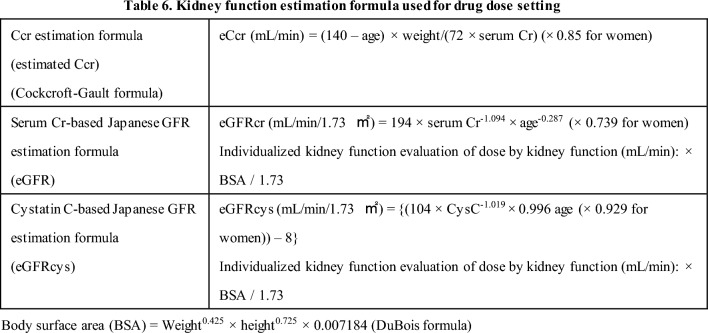


#### 11-7 Antiviral drug selection for CKD patients with herpesvirus infection

[Commentary summary]

Acyclovir, valacyclovir, and famciclovir, which are used for treatments of Herpes Simplex/Herpes Zoster Virus infection, are drugs extracted by kidney. Therefore, their blood concentration increases in CKD patients owing to excretion delay, and the dose reduction should be needed. Adverse events occur with a certain frequency despite reduced doses, thus careful monitoring is desirable after the administration. Amenamevir, which is a hepatically metabolized drug, does not require a drug administration design based on kidney function; however, as it is metabolized by cytochrome P450 (CYP) 3A, drug interactions should be considered while administrating it to CKD patients.

#### 11-8 Selection of analgesics for CKD patients with pain

[Commentary summary]

In terms of analgesic selection, dose, and duration for CKD patients, minimizing the dose and frequency of the drug while considering the occurrence of side effects according to the individual patient’s condition, is desirable. In this section, we provide an overview of the following analgesics:

1. Non-steroidal anti-inflammatory drugs (NSAIDs): it is desirable to pay attention to concomitant drugs and not to use them regularly. There is lack of clear evidence suggesting the safe use of particularly selective cyclooxygenase-2 inhibitors, like celecoxib for patients with CKD.
2. Acetaminophen: there is lack of clear evidence suggesting long-term safe use when combined with other drugs or used regularly.
3. Inflammatory skin extract from rabbits inoculated with vaccinia virus: there is a lack of large-scale studies on the use of this drug in patients with CKD.
4. Gabapentinoids: administration should begin at a low dose and attention should be paid to the occurrence of side effects.
5. Opioids: a high level of expertise is needed. Administration should begin at a low dose and attention should be paid to the occurrence of side effects.
6. Antiepileptic drugs, antidepressants, anxiolytics, central muscle relaxants: there is a lack of controlled trials that have examined the efficacy or side effects of these drugs in patients with CKD alone. Some drugs are to be carefully administered or are contraindicated for patients with CKD.

#### 11-9 Discontinuation of drugs during sick days in patients with CKD

[Commentary summary]

- Elderly and CKD patients have a high risk of AKI, and drug-induced AKI are likely to occur. The risk of AKI, including drug-induced AKI, increases during acute illness. Therefore, patients with CKD should promptly consult physicians and/or medical staff and receive appropriate treatment, including dose tapering and temporary drug withdrawal in sick days.
- Sick day rules have been proposed for various diseases and clinical conditions; however, the definitions of sick days and sick day rules for patients with CKD have not been established. In dehydrated states, kidney function reduces due to decreased blood pressure and renal plasma flow, leading to high risk of drug-induced acute kidney injury. Therefore, temporary drug withdrawal or dose tapering of renally excreted drugs should be considered.
- In dehydrated states, a risk of AKI due to NSAIDs administration and a risk of lactic acidosis by biguanides increase, respectively. Therefore, NSAIDs and biguanides should be considered their withdrawal in acute illness. In dehydrated conditions, drug withdrawal of SGLT2 inhibitors should be implemented to prevent ketoacidosis among patients with diabetes and/or CKD. Temporary drug withdrawal of SGLT2 inhibitors during sick days for the treatment of chronic heart failure should be considered by physicians and/or medical staff, according to the patient’s clinical conditions.
- In dehydrated states, a risk of AKI by the administration of diuretics and RA system inhibitors increases; however, drug withdrawal may exacerbate heart failure and increase CVD risk. Hence, physicians and/or medical staff should consider drug withdrawal according to the patient’s clinical conditions.
- Temporary drug withdrawal of active vitamin D (Vit. D) drugs may be considered in prominently anorexic or dehydrated states for prevention of the onset and exacerbation of hypercalcemia and AKI.

### Chapter 12. Pregnancy

#### 12-1 CKD affects pregnancy outcome

[Commentary summary]

CKD has an adverse impact on pregnancies (pregnancy outcomes: premature birth, fetal death, low birth weight, gestational hypertensive syndrome, cesarean section rate, neonatal intensive care unit (NICU) admission rate).

#### 12-2 CKD-complicated pregnancies impact the prognosis of maternal kidney function

[Commentary summary]

CKD-complicated pregnancies are known to negatively impact the prognosis of maternal kidney function.

#### 12-3 Antihypertensive drugs recommended for patients with CKD who are pregnant or wish to have children

[Commentary summary]

Methyldopa, labetalol, hydralazine, and (sustained-release) nifedipine and amlodipine are the first-line oral antihypertensive drugs in patients with CKD who are pregnant or wish to have children.

#### 12-4 Immunosuppressants that can be used during pregnancy and lactation in patients with CKD

[Commentary summary]

Adrenal corticosteroids, cyclosporine, tacrolimus, and azathioprine can be used depending on the pathological condition of the patient. Meanwhile, mizoribine and mycophenolate mofetil (MMF) are teratogenic, and should be discontinued or switched with another immunosuppressant at the time of planning for pregnancy or childbearing. Cyclophosphamide impacts fertility depending on the dose and age; hence, it is desirable to refrain from using cyclophosphamide in pregnant women.

### Chapter 13. Elderly CKD

#### 13-1 Perspective of elderly CKD

[Commentary summary]

It is difficult to accurately evaluate the number of pre-dialysis patients with CKD in Japan; however, the number was estimated to be 13.28 million people in 2005 and 14.80 million people in 2015. A reason for this increase is thought to be the aging of the population. Many elderly people are diagnosed with CKD due to decreased residual kidney function; however, whether the cause of this decrease is only aging, or kidney disease is also involved, is yet to be known.

#### 13-2 Elderly CKD management

[Commentary summary]

There are large individual differences in the symptoms of elderly patients with CKD. The decision on the test or treatment policies should be made after taking not only the disease, but also the QOL and vital prognosis, into consideration. The patient’s decision-making process should be shared with multiple professions.

#### 13-3 Elderly CKD treatment

[Commentary summary]

There is lack of evidence showing the need for age-based changes in treatment goals or treatments associated with CKD. As with general elderly care, the benefits and harms of treatment should be examined while making a judgment, especially for elderly patients with CKD. Decisions should be made while ensuring that the QOL is not impaired.

#### 13-4 [CQ] Is antihypertensive therapy recommended for hypertensive CKD patients aged 75 years or older to reduce office blood pressure to less than 150/90 mmHg?

[Recommendation]

It is recommended that office blood pressure be below 150/90 mmHg to suppress the progression of CKD and onset of CVD. [2C]

If there are no adverse events such as ischemic symptoms in the brain, heart, kidneys, etc., AKI, electrolyte abnormalities, hypotension-related symptoms (vertigo, dizziness), and if tolerability is judged to be present, then maintaining the blood pressure to an office blood pressure level below 140/90 mmHg is recommended. [2C]

### Chapter 14. Initiation of dialysis

#### 14-1 Timing of referral to nephrologist for appropriate initiation of renal replacement therapy

[Commentary summary]

To secure the time needed for selecting RRT (hemodialysis, peritoneal dialysis, kidney transplantation) and the preparation period for the selected RRT, it is important to refer the patient to a nephrologist or specialized medical institution when CKD stage G4 is reached.

#### 14-2 Significance of explanation and education about RRT by multiple disciplines

[Commentary summary]

It has been reported that multidisciplinary explanations and education about RRT are associated with slowing the progression of kidney injury, delaying the initiation of RRT, avoiding emergency dialysis, and selection of RRT.

#### 14-3 CVD screening at the time of dialysis initiation and kidney transplantation

[Commentary summary]

The risk of CVD onset increases as the CKD severity increases. It is desirable to conduct CVD screening at CKD stage G5, and prior to initiation of RRT.

### Chapter 15. Kidney transplantation

#### 15-1 Management of the living kidney donor after kidney donation

[Commentary summary]

Living donors after kidney donation are at risk of end-stage kidney disease. Therefore, it is important to conduct adequate follow-up as patients with CKD.

#### 15-2 [CQ] Is preemptive kidney transplantation (PEKT) recommended for patients desiring kidney transplantation?

[Recommendation]

It is suggested that preemptive kidney transplantation (PEKT) be conducted for patients desiring kidney transplantation. [2C]

#### 15-3 [CQ] Is kidney transplantation recommended as RRT for elderly patients with CKD?

[Recommendation]

Kidney transplantation is preferred as an RRT over dialysis therapy for elderly patients with CKD. [2C]

However, it should be limited to elderly patients who are expected to be at a low risk of early post-transplantation mortality. [None D]

#### 15-4 [CQ] Is kidney transplantation recommended as RRT for patients with diabetic kidney disease (DKD)?

[Recommendation]

Kidney transplantation is preferred as an RRT over dialysis therapy for patients with DKD. [2C]

### Chapter 16–1. Intractable diseases: IgA nephropathy.

#### 16-1-1 Natural course and prognosis of IgA nephropathy

[Commentary summary] The incidence of IgA nephropathy is reported to be 2.5 per 100,000 people annually; however, the incidence in Japan may be higher than that in other countries. In terms of renal prognosis, many studies have shown that the 10-year kidney survival rate is between 81 and 87%.

#### 16-1-2 Indicators related to prognosis of IgA nephropathy

[Commentary summary]

Important prognostic clinical indicators of IgA nephropathy are proteinuria and kidney function. Clinical severity classification (C-Grade) is defined by these factors. In addition, the prognostic indicators for histopathological findings of renal biopsy are crescent and glomerulosclerosis. Based on the ratio of these histological lesion, histological severity classification (H-Grade) is defined. The prognostic indicators of IgA nephropathy are assessed by both C-Grade and H-Grade. The Oxford classification (MEST-C score) and the International IgAN Prediction Tool are also used to predict prognosis. It is difficult to judge the disease activity of IgA nephropathy by uniform indicators. Here, we outline the prognostic indicators that are currently used clinically and pathologically.

#### 16-1-3 Treatment for IgA nephropathy

[Commentary summary]

The main therapeutic interventions for adult IgA nephropathy in Japan are RA system inhibitors (RAS-i), corticosteroids, immunosuppressants, tonsillectomy (+ steroid pulse therapy). General treatment for CKD also includes blood pressure management, salt intake reduction, lipid control, and smoking cessation guidance. However, with the exception of RAS-i and corticosteroids, there are insufficient comparative studies to validate the evidence. Therefore, clinical questions (CQs) of treatment in the “Evidence-based clinical practice guideline for IgA nephropathy 2020” targeted RAS-i and corticosteroids.

### Chapter 16–2 Intractable diseases. Nephrotic syndrome

#### 16-2-1 Measurement of serum anti-PLA2R antibodies for diagnosis of primary membranous nephropathy (MN) in adult nephrotic syndrome

[Commentary summary]

Measurement of serum anti-PLA2R antibodies is useful for diagnosis of primary MN in adult nephrotic syndrome. However, as of December 2023, this test is not covered by national health insurance; therefore, it is not recommended for all patients. These measurements may be done when conducting a kidney biopsy is difficult.

#### 16-2-2 Treatment for minimal change nephrotic syndrome (MCNS) in adults

[Commentary summary]

Steroid monotherapy should be selected for cases of initial onset, while dose tapering should be done 1–2 weeks after remission. For recurrent cases, steroids as well as cyclosporine should be concomitantly used. For frequently recurrent cases or steroid-dependent cases, the addition or change of immunosuppressants should be considered.

#### 16-2-3. Treatment for primary focal segmental glomerulosclerosis (FSGS) in adults

[Commentary summary]

High-dose oral prednisolone (PSL) equivalent to 1 mg/kg body weight per day (up to 60 mg per day) is used as initial treatment for FSGS with nephrotic syndrome. For recurrent cases following induction of remission, frequently recurrent cases, or steroid-dependent cases, the administration of immunosuppressants and rituximab should be considered according to the treatment for MCNS with frequent recurrence, or steroid dependence. Steroid-resistant FSGS should be treated with concomitant administration of steroids and cyclosporine.

#### 16-2-4 Treatment for primary membranous nephropathy (MN)

[Commentary summary]

MN presenting with nephrotic syndrome should be treated according to the treatment algorithm for primary nephrotic MN, which was developed as a part of the “Evidence-based clinical practice guideline for nephrotic syndrome 2020”, taking into account the results of a network meta-analysis conducted in 2019 and actual clinical practice in Japan.

### Chapter 16-3 Intractable diseases: polycystic kidney disease (PKD)

#### 16-3-1 Consultation with nephrologists and specialized medical institutions for autosomal dominant polycystic kidney disease (ADPKD) patients

[Commentary summary]

Patients should consult a medical institution after the detection of multiple cysts in the kidney by imaging tests such as abdominal ultrasound tests. ADPKD is a progressive disease in which kidney function decreases with age, and patients with suspected or diagnosed ADPKD should be treated in coordination with nephrologists. We provide commentary on patients where a consultation with a nephrologist is particularly recommended.

#### 16-3-2 Tolvaptan treatment in ADPKD patients

[Commentary summary]

To suppress kidney function deterioration, administration of tolvaptan therapy is recommended for adult patients with ADPKD that progresses rapidly or is expected to progress rapidly, under observation for adverse events accompanying diuresis and liver function test values.

#### 16-3-3 Antihypertensive therapy in ADPKD patients

[Commentary summary]

Hypertension is the most common complication in 50–80% of ADPKD patients. Antihypertensive therapy with ACE inhibitors or ARBs is expected to suppress proteinuria and prevent progression to end-stage kidney disease. Conducting strict antihypertensive therapy for a blood pressure of less than 110/75 mmHg in ADPKD patients aged < 50 years, having an eGFR > 60 mL/min/1.73 m^2^ requires caution against side effects such as dizziness and lightheadedness. However, such treatment has been shown to improve albuminuria, left heart hypertrophy, and increase the growth rate of total kidney volume. In particular, it can be expected to have a suppressive effect on eGFR decrease in Mayo Classification Class ID–IE patients who are expected to have rapid progression to kidney failure. Based on the above, antihypertensive therapy in ADPKD patients with hypertension should center on ACE inhibitors or ARBs and target a blood pressure of less than 140/90 mmHg according to CKD, or a blood pressure of less than 130/80 mmHg in proteinuria-positive patients. Furthermore, strict antihypertensive therapy of less than 110/75 mmHg is suggested only for patients aged less than 50 years, having an eGFR > 60 mL/min/1.73 m^2^, and who can tolerate such treatment.

### Chapter 16-4 Intractable diseases: rapidly progressive glomerulonephritis (RPGN)

#### 16-4-1 ANCA measurement in RPGN

[Commentary summary]

For differential diagnosis of RPGN, it is suggested that serum ANCA measurements be made with MPO-ANCA and PR3-ANCA using the enzyme immunoassay (EIA) method as the first-line method.

#### 16-4-2 Anti-glomerular basement membrane (GBM) antibody measurement in RPGN

[Commentary summary]

It is recommended that serum anti-GBM antibody measurements be made for the differential diagnosis of RPGN.

#### 16-4-3 Kidney biopsy in RPGN

[Commentary summary]

It is suggested that a kidney biopsy be conducted for differential diagnosis of RPGN.

### Chapter 17. Pediatric CKD

#### 17-1 Diagnosis of pediatric CKD (Table 7)

[Commentary summary]

The diagnostic criteria for pediatric CKD generally follow that for adults; however, the stage classification does not include the criteria for proteinuria and is classified according to GFR, which is physiologically low in children under 2 years of age. Therefore, the serum Cr level is used as an indicator to judge the stage based on the percentage of normal renal function in children of the same age. For detecting pediatric CKD, it is important to diagnose kidney dysfunction early by recognizing abnormal serum Cr levels, which differ according to age and sex.
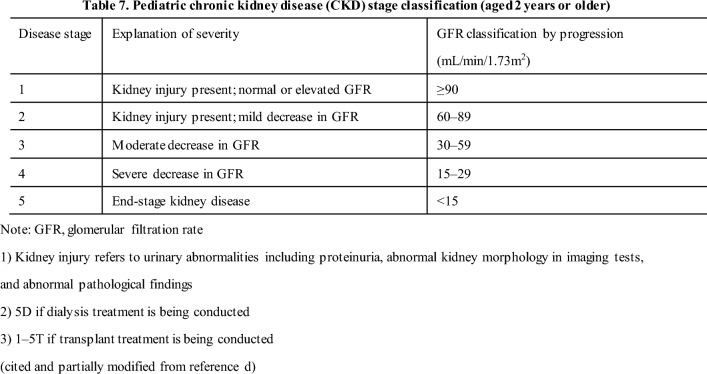


#### 17-2 Epidemiology of pediatric CKD

[Commentary summary]

The prevalence of pre-dialysis CKD (3 months–15 years old) of stage 3 or higher in Japan is calculated to be 2.98 out of 100,000 people. Non-glomerular diseases accounted for 91% of primary diseases, of which 68% were congenital anomalies of the kidney and urinary tract (CAKUT).

#### 17-3 Pediatric kidney disease screening

[Commentary summary]

There is a lack of reports with a high level of evidence for pediatric kidney disease screening; however, such screening may contribute to the early detection of pediatric patients with CKD and the early initiation of appropriate management, which may contribute to a reduction in renal mortality.

#### 17-4 [CQ] Is the use of RA system inhibitors recommended for pediatric CKD?

[Recommendation]

The oral administration of RA system inhibitors is suggested for pediatric CKD patients with proteinuria because it is expected to reduce proteinuria. [2C]

#### 17-5 Pediatric CKD and hypertension/CVD

[Commentary summary]

Complications of hypertension with pediatric CKD may be a risk factor for decreased kidney function. Additionally, pediatric CKD may be a risk factor for CVD, which is related to vital prognosis.

There have been few research reports with a high evidence level; however, strict management of hypertension associated with pediatric CKD may lead to an improvement in kidney function prognosis.

#### 17-6 Vaccination against pediatric CKD

[Commentary summary]

Pediatric CKD patients undergoing conservative treatment have reduced immune reactions to infection; hence, CKD may have a large impact on infection morbidity and mortality. Therefore, vaccination is an important strategy for disease management in these pediatric CKD patients. Meanwhile, the reduced post-vaccination antibody reaction and antibody duration in these patients have led to concerns about the immunogenicity and efficacy of vaccination in addition to safety concerns. Minimizing the risk of diseases in pediatric CKD patients that could be prevented with vaccines requires all the people caring for these patients to be aware of the recommended pediatric vaccination schedule and vaccination management in patients with pediatric CKD, including end-stage kidney disease.

#### 17-7 Lifestyle habits (obesity, exercise) in pediatric CKD

[Commentary summary]

Obesity is a factor in CKD progression and CVD onset, and it is an important issue in pediatric CKD patients. Adequate physical activity, adequate screen time (time spent watching TV or mobile phones, playing games, or using a computer), and sufficient sleep are likely to be effective in improving and preventing obesity in pediatric CKD patients. Additionally, though there is little evidence that exercise suppresses the progression of pediatric CKD or reduces proteinuria, it is highly likely that it contributes to improved QOL and motor function, and it is desirable to conduct moderate-intensity exercise for at least 30 min a day within the range that considers individual exercise tolerance and comorbidities.

#### 17-8 [CQ] Is protein intake restriction recommended for pediatric CKD patients?

[Recommendation]

The suppressive effect of protein intake restriction on progression of kidney dysfunction in pediatric CKD is unclear, and growth disorders may occur, so it is proposed that such restrictions not be conducted. [2B]

#### 17-9 Diet therapy (energy/salt) in pediatric CKD

[Commentary summary]

Appropriate energy intake is essential for achieving good growth and development. Therefore, in the current circumstances, where there is no high-quality evidence to support the usefulness of restricting energy intake, energy intake restrictions for pediatric CKD should not be conducted. Rather, if the pathological conditions or complications of CKD prevent the intake of energy needed for growth and development, an intake of an amount that exceeds the estimated energy requirement should be considered.

Salt intake restrictions for pediatric CKD should be considered according to both primary disease and CKD stage. Congenital anomalies of the kidney and urinary tract (CAKUT) cases may require salt intake supplementation rather than restriction. If pulmonary edema or hypertension are observed, then salt intake restrictions should be implemented.

#### 17-10 [CQ] Is human growth hormone administration recommended for pediatric CKD patients with growth disorders?

[Recommendation]

The administration of human growth hormone to pediatric CKD patients with growth disorders is recommended since its administration significantly improves height gain. [1B]

#### 17-11 Renal anemia in pediatric CKD

[Commentary summary]

There is no research with a high evidence level for renal anemia in pediatric CKD patients, so the policy for renal anemia in adult CKD patients is generally followed. However, it is necessary to recognize that pediatric CKD and adult CKD differ in their primary disease, differ in their drug dose and treatment response depending on physique, and differ in reference values and complications depending on age. Criteria for initiation of iron supplementation are pediatric CKD patients with anemia who are iron deficient [TSAT ≤ 20% or serum ferritin level ≤ 100 ng/mL (100 µg/L)]. Criteria for initiation of ESA therapy are when the Hb level is below 10 g/dL after multiple tests.

#### 17-12 Management of CKD-MBD in pediatric CKD

[Commentary summary]

CKD-mineral and bone disorder (CKD-MBD) is defined as the impact of bone and mineral metabolism on skeletal formation and calcification of blood vessels and soft tissue under the pathological condition of CKD, and it also impacts growth in children. In CKD-MBD management in pediatric CKD, it is recommended that the serum Ca level and P level management targets be within the range of reference values corresponding to age at all CKD stages. Hyperphosphatemia is controlled by phosphorus intake restrictions and the use of phosphate binders. Additionally, serum intact PTH management targets are ≤ 70 pg/mL up to CKD stage 3, ≤ 100 pg/mL for stage 4, and 100–300 pg/mL for stages 5 and 5D. Active vitamin D preparation administration is indicated for patients with high serum intact PTH levels without hypercalcemia.

#### 17-13 Pediatric CKD health care transition

[Commentary summary]

“Health care transition is the process of transitioning from pediatric to adult health care, regardless of transfer”, and transfer is only a partial event of transition. It is important that support be provided for the independence (autonomy) of the patient themselves even if transfer is not conducted. The transition program helps children with chronic diseases make a smooth transition from pediatric care to adult care and systematically supports patients’ independence/autonomy and social participation. It is a program that supports the growth of patients toward independence/autonomy, and it is desirable to start support with a specific eye toward transition from around the age of 12 years. Pediatric CKD often has child-specific diseases and complications and the treatment policy and management environment for each disease often differ between children and adults. There is a need to take the time to explain to the patient and obtain their understanding before transferring to an adult clinic. Additionally, at the time of transfer, the pediatrician and the adult clinician should share information, and sufficient cooperation between the two clinical departments is essential.

#### 17-14 Initiation of renal replacement therapy

[Commentary summary]

For the initiation of RRT, physicians who specialize in pediatric kidney disease should provide sufficient information to the pediatric CKD patients and their guardians, and consider prior examinations, pathological condition of the pediatric CKD patient, family circumstances, and their wishes, after which they should comprehensively determine the renal replacement therapy modality (peritoneal dialysis, hemodialysis, kidney transplantation) and timing. The initiation of renal replacement therapy requires a preparatory period, so a referral to a facility for initiating renal replacement therapy is desirable when kidney function has decreased to around 30 mL/min/1.73 m^2^ and progression to end-stage kidney disease is unavoidable in the future.

#### 17-15 [CQ] Is preemptive kidney transplantation (PEKT) recommended in pediatric CKD patients?

[Recommendation] It is suggested that PEKT be conducted in pediatric CKD patients since its implementation may improve the kidney transplantation survival rate compared to kidney transplantation after dialysis. [2D]

**List of major abbreviation**

**Table:** 

Abbreviations	English
ACE	Angiotensin converting enzyme
ADPKD	Autosomal dominant polycystic kidney disease
AKI	Acute kidney injury
ANCA	Anti-neutrophil cytoplasmic antibody
ARB	Angiotensin II receptor blocker
Ccr	Creatinine clearance
CKD	Chronic kidney disease
CKD-MBD	CKD-mineral and bone disorder
CQ	Clinical question
Cr	Creatinine
CVD	Cardiovascular disease
DKD	Diabetic kidney disease
DM	Diabetes mellitus
eGFR	Estimated glomerular filtration rate
ESA	Erythropoiesis stimulating agent
GFR	Glomerular filtration rate
HIF-PH	Hypoxia-inducible factorprolyl hydroxylase
JSN	Japanese Society of Nephrology
KDIGO	Kidney Disease: Improving Global Outcomes
MN	Membranous nephropathy
PKD	Polycystic kidney disease
PLA2R	Phospholipase A2 receptor
PPI	Proton pump inhibitor
PTH	Parathyroid hormone
QOL	Quality of life
RA	Renin–angiotensin
RPGN	Rapidly progressive glomerulonephritis
RRT	Renal replacement therapy
SGLT2	Sodium–glucose cotransporter 2
TSAT	Transferrin saturation

**Conflict of Interest Disclosures:**

**Honoraria:** Shoichi Maruyama (AstraZeneca, Bayer Yakuhin, Ltd., Mitsubishi Tanabe Pharm, Alexion Pharmaceuticals, Inc., Astellas Pharma Inc., Kyowa Kirin Co., Ltd., Ono Pharmaceutical Co., Ltd., Torii Pharmaceutical Co., Ltd.), Shinji Kume (Kyowa Kirin Co., Ltd., Mitsubishi Tanabe Pharm, AstraZeneca, Daiichi Sankyo, Boehringer Ingelheim, Eli Lilly), Hirokazu Okada (Kyowa Kirin, Daiichi Sankyo, AstraZeneca Pharm., Torii Pharm., Mitsubishi Tanabe Pharm, Ono Pharm., Astellas Pharm., Boehringer Ingelheim), Tsuneo Konta (Bayer Yakuhin, Ltd., Mitsubishi Tanabe Pharm, Mochida Pharmaceutical Co., Ltd., AstraZeneca Pharm), Hitoshi Suzuki (Kyowa Kirin Co., Ltd, Mochida Pharmaceutical Co., Ltd.), Tetsuhiro Tanaka (Astellas, Kyowa Kirin Co., Ltd., Mitsubishi Tanabe Pharm, Torii, Bayer Yakuhin, Ltd.), Naotake Tsuboi (AstraZeneca, Kissei Pharmaceutical Co., Ltd.), Naoki Nakagawa (AstraZeneca), Kei Fukami (AstraZeneca, Boehringer Ingelheim, Mitsubishi Tanabe Pharma, Ono Pharm.), Keitaro Yokoyama (Torii Pharmaceutical Co., Ltd., Ono Pharm., Kyowa Kirin Co., Ltd.), Jun Wada (AstraZeneca, Bayer Yakuhin, Ltd., Boehringer Ingelheim, Daiichi Sankyo, Kyowa Kirin Co., Ltd., Novo Nordisk, Mitsubishi Tanabe Pharm), Katsuhiko Asanuma (Kyowa Kirin Co., Ltd., AstraZeneca), Koichi Asahi (Mochida Pharmaceutical Co., Ltd.), Masanori Abe(Astellas Pharma Inc., Bayer Yakuhin, Ltd., Kyowa Kirin Co., Ltd., Mitsubishi Tanabe Pharma Corporation, Torii Pharmaceutical Co., Ltd.), Daiji Kawanami (Mitsubishi Tanabe Pharm, Novo Nordisk, Sanofi, Sumitomo Pharma Co., Ltd., Bayer Yakuhin, Ltd., Daiichi-Sankyo), Hirotaka Komaba (Kissei Pharmaceutical, Kyowa Kirin Co., Ltd., Sanwa Kagaku Kenkyusho), Kenei Sada (Glaxo Smith Kline K.K.), Tadashi Sofue (AstraZeneca, Mitsubishi Tanabe Pharma.), Hideki Fujii (Sanofi, Astrazeneca, Kissei Pharma Co., Ltd., Kyowa Kirin Co., Ltd., Sumitomo Pharma, Bayer Yakuhin, Ltd.), Junichi Hoshino (AstraZeneca, Astellas, Kissei, Kyowa-Kirin, Mitsubishi Tanabe Pharm), Michihiro Hosojima (AstraZeneca),

Yoshinari Yasuda (Astellas Pharma Inc., AstraZeneca, Kowa Company, Ltd., Mochida Pharmaceutical Co., Ltd.).

**Manuscript fees:** Shinya Nakatani (Otsuka Pharmaceutical Co., Ltd.).

**Research funding:** Shoichi Maruyama (Chugai Pharmaceutical Co., Ltd., Ono Pharmaceutical Co., Ltd., Mitsubishi Tanabe Pharma Co., Ltd., Rohto Pharmaceutical Co., Ltd.), Shinji Kume (Boehringer Ingelheim), Yoshitaka Isaka (AstraZeneca, Kyowa Kirin, Otsuka Pharmaceutical, Mitsubishi Tanabe Pharma Corporation, Astellas), Hirokazu Okada (Kyowa Kirin Co., Ltd., Torii Pharm.), Naotake Tsuboi (Otsuka Pharmaceutical Co., Ltd., Sanofi Co., Ltd., Zenyaku Holdings Co., Ltd.), Saori Nishio (Rege Nephro Co., Ltd., Otsuka Pharmaceutical Co., Ltd., Kyowa Kirin Co., Ltd.), Kei Fukami (Boehringer Ingelheim), Katsuhiko Asanuma (Kyowa Kirin Co.), Daiji Kawanami (Tanabe-Mitsubishi), Hirotaka Komaba (Kissei Pharmaceutical, Kyowa Kirin, Sanwa Kagaku Kenkyusho), Hirotaka Komaba (Kyowa Kirin Co., Ltd.), Atsuko Nakatsuka (Mitsubishi Tanabe pharma Co.), Hideki Fujii (JCR Pharma, Tsumura), Junichi Hoshino (Otsuka Pharm.), Michihiro Hosojima (Kameda Seika CO., LTD., Sato Foods Co., LTD., Forica Foods Co., Ltd., Biotech Japan Corporation), Tomoki Kosugi (Chugai Pharmaceutical Co., Ltd., Ono Pharmaceutical Co., Ltd., Mitsubishi Tanabe Pharma Co., Ltd., Rohto Pharmaceutical Co., Ltd.).

**Subsidies or Donations:** Shoichi Maruyama (Otsuka Pharmaceutical Co., Ltd., Kyowa Kirin Co., Ltd., Sumitomo Pharma Co., Ltd., Torii Pharmaceutical Co., Ltd.), Shinji Kume (Kyowa Kirin, Mitsubishi Tanabe Pharm, Kowa, Sumitomo Pharma, Daiichi Sankyo, Boehringer Ingelheim, Novo Nordisk, Nipro, Takeda Pharmaceuticals, Teijin Pharma, MSD), Hirokazu Okada (Kyowa Kirin, Torii Pharm.), Naotake Tsuboi (Bayer Yakuhin, Ltd, Chugai Pharmaceutical Co., Ltd., Kyowa Kirin Co., Ltd. Otsuka Pharmaceutical Co., Ltd., Sumitomo Pharma Co., Ltd., Torii Pharmaceutical Co., Ltd.),

Naoki Nakagawa (Otsuka Pharmaceutical Co., Ltd., Daiichi Ssankyo Co., Ltd.), Jun Wada (Bayer, Chugai, Kyowa Kirin, Otsuka Pharmaceutical Co., Ltd., Shionogi, Sumitomo Pharma Co., Ltd., Mitsubishi Tanabe Pharm), Takashi Wada (Bayer Yakuhin, Ltd.), Masanori Abe (Kyowa Kirin Co., Ltd., Mitsubishi Tanabe Pharma, Torii Pharmaceutical Co., Ltd.), Daiji Kawanami (Boehringer Ingelheim, Sumitomo Pharma Co., Ltd., Bayer, Nipro), Hideki Fujii (Kyowa Kirin Co., Ltd., Roche Diagnostics, Bayer Yakuhin, Ltd., Chugai Pharmaceutical Co., Ltd.), Hideo Yasuda (Otsuka Pharmaceutical Co., Ltd.), Tomoki Kosugi (Otsuka Pharmaceutical Co., Ltd., Kyowa Kirin Co., Ltd., Sumitomo Pharma Co., Ltd., Torii Pharmaceutical Co., Ltd.).

**Endowed departments by commercial entities:** Shoichi Maruyama (Baxter Ltd.),

Michihiro Hosojima (Kameda Seika Co., Ltd.).

**List of Contributors**

Shoichi Maruyama^1^, Eiichiro Kanda^2^, Shinji Kume^3^, Yoshitaka Isaka^4^, Kenji Ishikura^5^, Joichi Usui^6^, Keiko Uchida^7^, Hirokazu Okada^8^, Tsuneo Konta^9^, Chie Saito^10^, Hitoshi Suzuki^11^, Tetsuhiro Tanaka^12^, Naotake Tsuboi^13^, Naoki Nakagawa^14^, Saori Nishio^15^, Kei Fukami^16^, Hirokazu Honda^17^, Kosuke Masutani^18^, Keitaro Yokoyama^19^, Jun Wada^20^, Takashi Wada^21^, Takehiko Wada^22^, Katsuhiko Asanuma^23^, Koichi Asahi^24^, Masanori Abe^25^, Takuji Ishimoto^26^, Daiji Kawanami^27^, Hirotaka Komaba^28^, Kenei Sada^29^, Tadashi Sofue^30^, Shinya Nakatani^31^, Atsuko Nakatsuka^32^, Satoshi Hibino^33^, Hideki Fujii^34^, Junichi Hoshino^35^, Michihiro Hosojima^36^, Akito Maeshima^37^, Yukio Maruyama^38^, Takahito Moriyama^39^, Hideo Yasuda^40^, Yoshinari Yasuda^1^, Suguru Yamamoto^41^, Tomoki Kosugi^1^

Department of Nephrology, Nagoya University Graduate School of Medicine^1^

Medical Science, Kawasaki Medical School^2^

Department of Medicine, Shiga University of Medical Science^3^

Department of Nephrology, Osaka University Graduate School of Medicine^4^

Department of Pediatrics, Kitasato University School of Medicine^5^

Department of Nephrology, Institute of Medicine, University of Tsukuba^6^

Yokosuka Clinic^7^

Department of Nephrology, Saitama Medical University^8^

Department of Public Health and Hygiene, Yamagata University Graduate School of Medical Science^9^

Department of Nephrology, Faculty of Medicine, University of Tsukuba^10^

Department of Nephrology and Hypertension, Juntendo University Urayasu Hospital^11^

Department of Nephrology, Rheumatology and Endocrinology, Tohoku University Graduate School of Medicine^12^

Department of Nephrology, Fujita Health University School of Medicine^13^

Division of Cardiology and Nephrology, Department of Internal Medicine, Asahikawa Medical University^14^

Rheumatology and Nephrology, Hokkaido University Hospital^15^

Division of Nephrology, Department of Medicine, Kurume University School of Medicine^16^

Department of Nephrology, Showa University School of Medicine^17^

Division of Nephrology and Rheumatology, Department of Internal Medicine, Faculty of Medicine, Fukuoka University^18^

Jikei University Harumi Toriton Clinic^19^

Department of Nephrology, Rheumatology, Endocrinology and Metabolism, Okayama University Graduate School of Medicine, Dentistry and Pharmaceutical Sciences^20^

Kanazawa University^21^

Department of Nephrology, Toranomon Hospital^22^

Department of Nephrology, Chiba University Graduate School of Medicine^23^

Division of Nephrology and Hypertension, Department of Internal Medicine, Iwate Medical University School of Medicine^24^

Division of Nephrology, Hypertension and Endocrinology, Department of Medicine, Nihon University School of Medicine^25^

Department of Nephrology and Rheumatology, Aichi Medical University^26^

Department of Endocrinology & Diabetes, Fukuoka University School of Medicine^27^

Division of Nephrology, Endocrinology and Metabolism, Tokai University School of Medicine^28^

Department of Clinical Epidemiology, Kochi Medical School^29^

Department of Cardiorenal and Cerebrovascular Medicine, Faculty of Medicine, Kagawa University^30^

Department of Metabolism, Endocrinology and Molecular Medicine, Osaka Metropolitan University Graduate School of Medicine^31^

Division of Kidney, Diabetes and Endocrine Diseases, Okayama University Hospital^32^

Pediatric Nephrology, Aichi Children's Health and Medical Center^33^

Division of Nephrology and Kidney Center, Kobe University Graduate School of Medicine^34^

Department of Nephrology, Tokyo Women's Medical University^35^

Department of Clinical Nutrition Science, Kidney Research Center, Niigata University Graduate School of Medical and Dental Sciences^36^

Department of Nephrology and Hypertension, Saitama Medical Center, Saitama Medical University^37^

Division of Nephrology and Hypertension, Department of Internal Medicine, The Jikei University School of Medicine^38^

Department of Nephrology, Tokyo Medical University^39^

First Department of Medicine, Hamamatsu University School of Medicine^40^

Division of Clinical Nephrology and Rheumatology, Niigata University Graduate School of Medical and Dental Sciences^41^

